# Experimental co-infection of variant infectious bursal disease virus and fowl adenovirus serotype 4 increases mortality and reduces immune response in chickens

**DOI:** 10.1186/s13567-021-00932-y

**Published:** 2021-04-29

**Authors:** A-hui Xu, Lu Sun, Kai-hang Tu, Qing-yuan Teng, Jia Xue, Guo-zhong Zhang

**Affiliations:** Key Laboratory of Animal Epidemiology of the Ministry of Agriculture, College of Veterinary Medicine, China Agricultural University, Beijing, 100193 China

**Keywords:** Infectious bursal disease virus, Fowl adenovirus serotype 4, Co-infection, Pathogenicity

## Abstract

Infectious bursal disease virus (IBDV) and fowl adenovirus serotype 4 (FAdV-4) cause infectious bursal disease (IBD) and hydropericardium-hepatitis syndrome, respectively. Recently, studies have reported co-infections of poultry with IBDV and FAdV-4, which is an important problem in the poultry industry. Here, the variant IBDV strain ZD-2018-1 and FAdV-4 isolate HB1501 were used to assess the pathogenicity of co-infection in 1-day-old specific pathogen-free (SPF) chickens. Compared with chickens infected with only FAdV-4, those coinfected with IBDV and FAdV-4 showed enhanced clinical symptoms, higher mortality, more severe tissue lesions, and higher biochemical index levels. Furthermore, the expression of interleukin (IL)-6, IL-1β, and interferon-γ mRNAs in the IBDV-FAdV-4 coinfected chickens was delayed, and the antibody response levels were significantly lower in those birds compared with the FAdV-4-infected chickens. These results indicate that co-infection with variant IBDV ZD-2018-1 and FAdV-4 HB1501 could significantly promote the pathogenicity of FAdV-4 and reduce the immune response in chickens. This study provides the foundation for further investigation of the interaction mechanism in IBDV and FAdV-4 co-infection.

## Introduction

Infectious bursal disease virus (IBDV) is an icosahedral, non-enveloped, double-stranded RNA virus belonging to the genus *Avibirnavirus*, in the family Birnaviridae, which can cause an acute, highly contagious, immunosuppressive disease known as infectious bursal disease (IBD) [1, 2]. This disease of young chickens is characterized mainly by gelatinous material exudation, atrophy, or hemorrhage of the bursa of Fabricius (BF) [3]. The damage to the BF impairs the immune function of infected chickens, leading to immunosuppression and resulting in increased susceptibility to secondary infection and vaccination failure [4]. IBDV has two serotypes. Serotype I viruses are pathogenic to chickens, whereas serotype II viruses are not. Serotype I IBDV can be classified into four phenotypes: antigenic variant strains, classical strains, attenuated strains, and highly virulent strains [5]. Recently, a novel antigenic variant IBDV has been prevalent in eastern China [6, 7]. Notably, the present commercial IBDV vaccine cannot provide protect against this novel variant IBDV [6, 8], which poses a new challenge for the poultry industry.

Fowl adenovirus (FAdV), is an icosahedral, non-enveloped, double-stranded DNA virus belonging to the genus *Aviadenovirus* in the family Adenoviridae [9]. Aviadenoviruses can classified into five species, FAdV-A to FAdV-E, and 12 serotypes, FAdV-1 to -8a and FAdV-8b to -11 [10]. Infection with FAdVs can cause various clinical symptoms, including gizzard erosion, inclusion body hepatitis, and hydropericardium-hepatitis syndrome [11]. FAdV-4 was first reported in Angara Goth, Pakistan in 1987 and subsequently spread worldwide [12], causing huge economic losses to the poultry industry. Since 2015, FAdV-4 has occurred in several provinces in China [11, 13–15] and attracted much attention in the poultry industry. Furthermore, some studies have reported that poultry can become coinfected with FAdV and other avian diseases, such as avian influenza virus (AIV), IBDV, and chicken infectious anemia virus (CIAV) [16–19]. FAdV co-infection with other infectious agents has become a non-negligible issue.

The interactions between FAdV, the coinfecting agents, and their hosts need to be further investigated. Previous research has shown that highly virulent IBDV can enhance the pathogenicity of inclusion body hepatitis virus (IBHV) [20]. The objective of this study was to investigate the interaction mechanism between FAdV, IBDV, and their host by examining specific pathogen-free (SPF) chickens coinfected with the currently circulating novel variant IBDV and the epidemic FAdV-4 isolate.

## Materials and methods

### Cell line, animals, and ethics statement

A male leghorn hepatoma cell line (LMH) was kept in our laboratory. One-day-old and 3-week-old SPF chickens were purchased from the Beijing Boehringer Ingelheim Vital Biotechnology Co., Ltd. (China) and housed in isolators at China Agricultural University. All animal experiments were approved by Beijing Administration Committee of Laboratory Animals under the leadership of the Beijing Association for Science and Technology (approval ID SYXK [Jing] 2018-0038). The protocols for this experiment were approved by the Animal Welfare and Ethical Censor Committee at China Agricultural University.

### Viruses

The IBDV strain ZD-2018-1 (IBDV-ZD-2018-1) was propagated in SPF chickens infected orally, and the titer of IBDV-ZD-2018-1 in 3-week-old SPF chickens was 10^4.2^ the median chicken infectious dose (CID_50_)/0.2 mL [7]. The FAdV-4 strain HB1501 (FAdV-4-HB1501) was reproduced in LMH cells grown at 37 °C and was titrated by 50% tissue culture infection dose (TCID_50_) in LMH cells [21]. The titer of FAdV-4-HB1501 was 10^7.5^ TCID_50_/0.1 mL.

### Experimental design

A total of 140 1-day-old SPF chickens were randomly divided into four groups of 35 birds each: control group, FAdV-only, IBDV-FAdV co-infection, and IBDV-only. At 1 day of age, the chickens in the IBDV-FAdV co-infection or IBDV-only groups were orally inoculated with 200 µL of 10^3^ CID_50_ of IBDV-ZD-2018-1 while those in the control or FAdV-only groups were orally inoculated with 200 µL of normal saline solution. At 14 days of age, the chickens in the FAdV-only or IBDV-FAdV co-infection groups were challenged with 200 µL of 10^6^ TCID_50_ of FAdV-4-HB1501 via the oral route, while those in the control or IBDV-only groups were inoculated with 200 µL of normal saline solution via the same route. Ten chickens in each group were marked for clinical observation, and the remainder was used for sample collection. Food and water were provided ad libitum throughout the study. The 10 marked chickens from each group were observed daily for clinical signs over the 14 days following FAdV infection. Symptoms were scored as described in a previous study [7]. At 1, 3, 5, and 7 days post-infection (dpi) with FAdV-4-HB1501, three birds randomly selected from each group were euthanized for necropsy and physical examination. The liver, spleen, kidneys, thymus, BF, and duodenum were collected for use in virus detection and temporarily stored at −80 °C. Samples of the tissues described above were also preserved in 10% neutral formalin for histopathological examination. Cloacal swabs were collected for the detection of viral shedding. Serum samples were collected for the detection of biochemical indices.

### Histopathology test

Tissue samples fixed in 10% neutral formalin were processed routinely, embedded in paraffin, cut into 5-µm sections, and stained with hematoxylin and eosin. The microscopic lesions of the tissue sections were then observed under a light microscope. The lesions at 5 dpi were scored according to the severity of the histopathology change. Samples with a lack of obvious lesions were scored as 0, while those with slight, moderate, and severe lesions were scored as 1, 2, and 3, respectively.

### FAdV detection with RT-qPCR

Total DNA was extracted from the tissues and cloacal swabs described above using a TIANamp Genomic DNA Kit (Tiangen Biotech, Beijing, China) in accordance with the manufacturer’s instructions. The obtained DNA was used to quantify the viral load by RT-qPCR. Primers were designed based on conserved regions of the FAdV genome using Primer Premier 5.0 (N-F: 5′AAAACTGAGACTTTCCCACAA 3′; N-R: 5′AGATACCCTCCGAAGAACTAC 3′), as described by Li et al. [17]. All samples were tested in triplicate, and assays were repeated at least twice. The FAdV load was calculated from a standard curve.

### Expression assessment of immune-related genes by RT-qPCR

The spleens collected as described above were processed using an Animal Total RNA Isolation Kit (FOREGENE, Chengdu, China) following the manufacturer’s instructions. Approximately 500 ng of RNA was reverse-transcribed with PrimeScript™ RT Master Mix (Takara, Beijing, China) at 37 °C for 15 min, 85 °C for 5 s in accordance with the manufacturer’s instructions. To evaluate the cytokine gene expression, primers for chicken β-actin, interleukin (IL)-6, IL-1β, interferon (IFN)-α, and IFN-γ (Table 1) described in previous studies were used [22–24]. RT-qPCR for each gene was performed in a 20-µL volume of mixture composed of 2 × M5 HiPer SYBR Premix EsTaq (with Tli RNaseH) (Mei5bio, Beijing, China), primers, cDNA, and nuclease-free water. The RT-qPCR assay was conducted on a LightCycler® 96 Real-Time PCR system (Roche, Basel, Switzerland) with the following procedure: preincubation at 95 °C for 30 s, followed by 40 cycles of 95 °C for 5 s, 60 °C for 20 s. The analysis of the melting curve was performed as follows: 95 °C for 10 s, 65 °C for 60 s, and 97 °C for 1 s. The Ct values of each gene were normalized against β-actin, and the relative expression levels were determined using the 2^–∆∆Ct^ method.

**Table 1:** **Sequences of primers used in this study**

Primer name	Nucleotide sequences	References
β-actin	CAACACAGTGCTGTCTGGTGGTA	[21]
	ATCGTACTCCTGCTTGCTGATCC	
IL-6	CCAGAAATCCCTCCTCGCCAATC	[23]
	GCCCTCACGGTCTTCTCCATAAAC	
IL-1β	TCATCTTCTACCGCCTGGAC	[22]
	GTAGGTGGCGATGTTGACCT	
IFN-α	ATGCCACCTTCTCTCACGAC	[22]
	AGGCGCTGTAATCGTTGTCT	
IFN-γ	ACACTGACAAGTCAAAGCCGC	[21]
	AGTCGTTCATCGGGAGCTTG	

### Biochemical assays

Biochemical indices in serum samples, including alanine aminotrasferase (ALT), aspartic aminotransferase (AST), lactic dehydrogenase (LDH), and urea, were detected in the test center of China Agricultural University Veterinary Teaching Hospital using a Roche Cobas c 501 chemistry analyzer (Roche).

### Serology

To determine the humoral immune response levels, serum samples were collected from the surviving chickens in each group at 7 days post-co-infection. Commercial ELISA kits were used for the detection of IBDV- (IDEXX Laboratories, Westbrook, ME, USA) and FAdV-specific antibodies (BioChek, South San Francisco, CA, USA) in accordance with the manufacturer’s instructions.

### Statistical analysis

Statistical analyses of RT-qPCR, biochemical index, and antibody level data were performed using GraphPad Prism version 6.01 (GraphPad Software Inc., San Diego, CA, USA). Statistical analysis was performed by using a two-way analysis of variance. Differences were considered statistically significant at *p* < 0.05.

## Results

### Clinical signs and gross lesions

**Figure 1:**
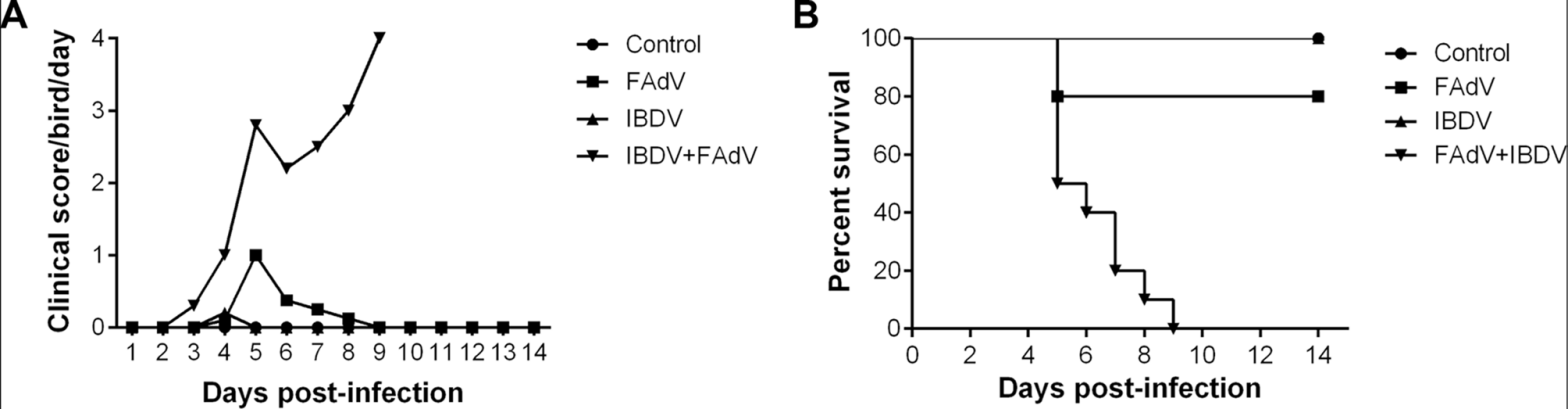
**Clinical scores and survival rates of infected chickens.** Clinical scores (**A**) and percentage survival (**B**) of chickens challenged with variant IBDV-ZD-2018–1 and/or FAdV-4-HB1501.

To understand the influence of IBDV infection on a subsequent infection with FAdV, the clinical signs scores were calculated over the 14 days following secondary infection. Chickens inoculated with FAdV-4-HB1501 alone (FAdV-only group) showed clinical symptoms beginning at 4 dpi. These symptoms peaked at 5 dpi, after which the chickens recovered gradually. Two chickens (20%) in this group died during the experimental period. The chickens in the IBDV-FAdV co-infection group began to show clinical symptoms at 3 dpi with FAdV-4-HB1501, and all chickens in this group died by 9 dpi with FAdV-4-HB1501. The chickens in the FAdV-only group or the IBDV-FAdV co-infection group showed similar signs, including ruffled feathers, depression, and prostration. There were no obvious clinical symptoms or death in the IBDV-only group or the control group (Figure 1).

At necropsy conducted 5 dpi with FAdV-4-HB1501, the liver of chickens in the FAdV-only group turned green. In the IBDV-only group, at 19 days post-challenge with IBDV-ZD-2018-1, the chickens showed obvious BF atrophy. Chickens in the IBDV-FAdV co-infection group, at 5 dpi with FAdV-4-HB1501, showed liver necrosis and atrophy of the thymus and BF. No gross lesions were observed in control group chickens.

### Histopathology

**Figure 2:**
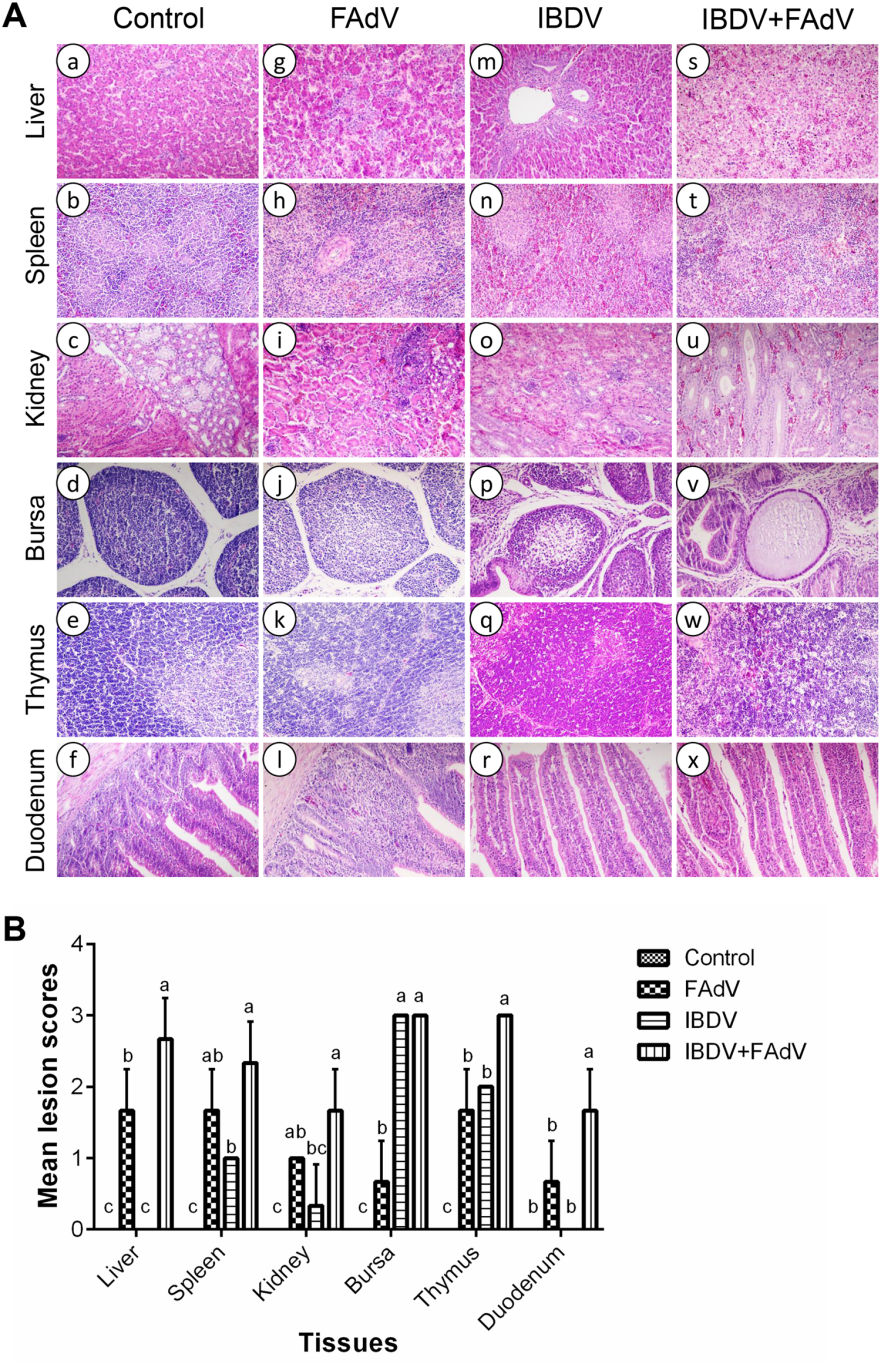
**Histopathologic changes and mean lesion scores in different groups.** **A** Representative images of hematoxylin and eosin-stained liver, spleen, kidney, bursa, thymus, and duodenum sections from chickens in the control (a–f, respectively), FAdV-only (g–l, respectively), IBDV-only (m–r, respectively), or IBDV-FAdV co-infection (s–x, respectively) groups at 5 dpi with FAdV-4-HB1501. Magnification: ×400. **B** Mean lesion scores in these organs at 5 dpi; ^a–c^Bars with no common superscript are significantly different (*p* < 0.05).

Microscopic lesions were examined in the liver, spleen, kidneys, thymus, BF, and duodenum of the infected chickens at 5 dpi with FAdV-4-HB1501. No obvious lesions were observed in the control group (Figure 2A, panels a–f). In the FAdV-only group, chickens showed moderate hepatocellular necrosis; lymphocyte reduction in the spleen, BF, and thymus; a small amount of lymphocyte infiltration in the renal tubulointerstitium; and slight hemorrhage in the duodenum (Figure 2A, panels g–l). In the IBDV-only group, obvious atrophy and lymphocyte necrosis in the follicle of the BF and lymphocyte reduction and necrosis in the spleen and thymus were observed (Figure 2A, panels m–r). In the IBDV-FAdV co-infection group, there were severe lesions in various organs, including a large area of hepatocellular necrosis; congestion in the kidney; a large amount of lymphocyte necrosis in the spleen, BF, and thymus; and severe necrosis of duodenum mucosa epithelium (Figure 2A, panels s–x). The mean lesion scores of various tissues in the different groups at 5 dpi are shown in Figure 2B.

### Viral DNA detection in tissues

**Figure 3:**
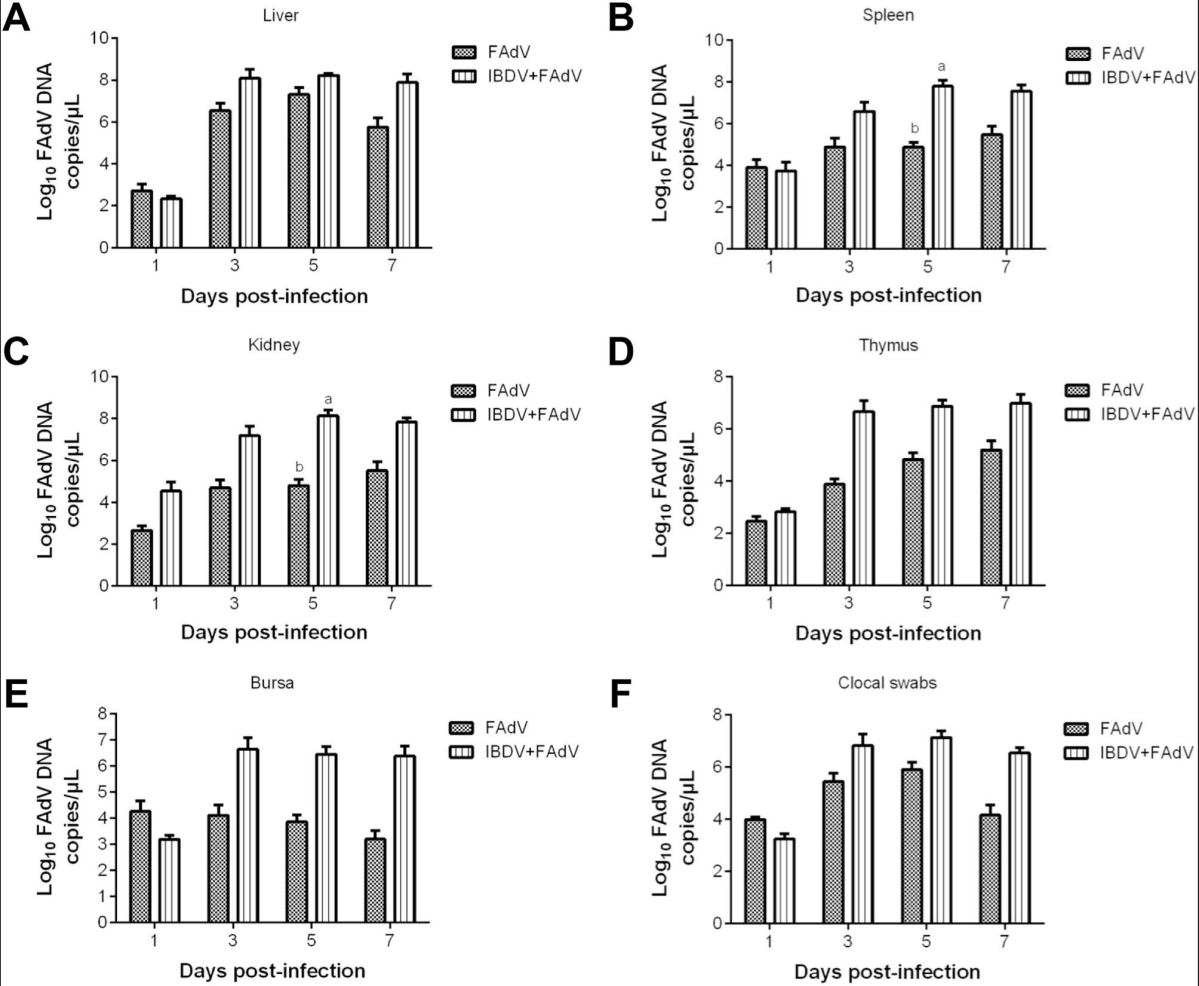
**Tissue viral loads of FAdV and cloacal swabs of chickens in different groups.** Chickens were inoculated with variant IBDV-ZD-2018–1 and/or FAdV-4-HB1501. Cloacal swabs were performed, and the viral loads of FAdV in various tissues were measured. ^a,b^Bars with different superscripts are significantly different (*p* < 0.05).

The viral loads in different tissues from the FAdV-only and IBDV-FAdV co-infection groups are shown in Figure 3. The FAdV genome copy numbers in the livers of these two groups peaked at 5 dpi with FAdV-4-HB1501, and the viral loads of the IBDV-FAdV co-infection group were higher than those of the FAdV-only group (Figure 3A). The viral loads in the spleens and kidneys from the co-infection group peaked at 5 dpi with FAdV-4-HB1501 while in the FAdV group the viral loads reached the maximum at 7 dpi. The viral copy numbers of the IBDV-FAdV co-infection group were higher than those of the FAdV-only group, with a statistically significant difference at 5 dpi (Figures 3B and C; *p* < 0.05). The viral load in the thymus of these two groups gradually increased and peaked at 7 dpi with FAdV-4-HB1501, and the viral copy numbers of the IBDV-FAdV co-infection group trended higher than those of the FAdV-only group, but this difference failed to reach statistical significance (Figure 3D). The maximum viral load in the BF of the FAdV-only and IBDV-FAdV co-infection group peaked at 1 and 3 dpi with FAdV-4-HB1501, respectively (Figure 3E). Last, the viral loads in the BF samples from the IBDV-FAdV co-infection group were higher than those in the FAdV-only group at most timepoints.

### Detection of viral shedding

Cloacal FAdV shedding was examined, and the results are shown in Figure 3F. The viral shedding of the FAdV-only and IBDV-FAdV co-infection groups each peaked at 5 dpi with FAdV-4-HB1501, after which they decreased. The level of viral shedding in chickens from the IBDV-FAdV co-infection group was considerably higher than that in the FAdV-only group at most timepoints, which is consistent with the viral loads measured in tissue samples from these two groups (Figures 3A–E).

### Cytokine mRNA expression level

**Figure 4:**
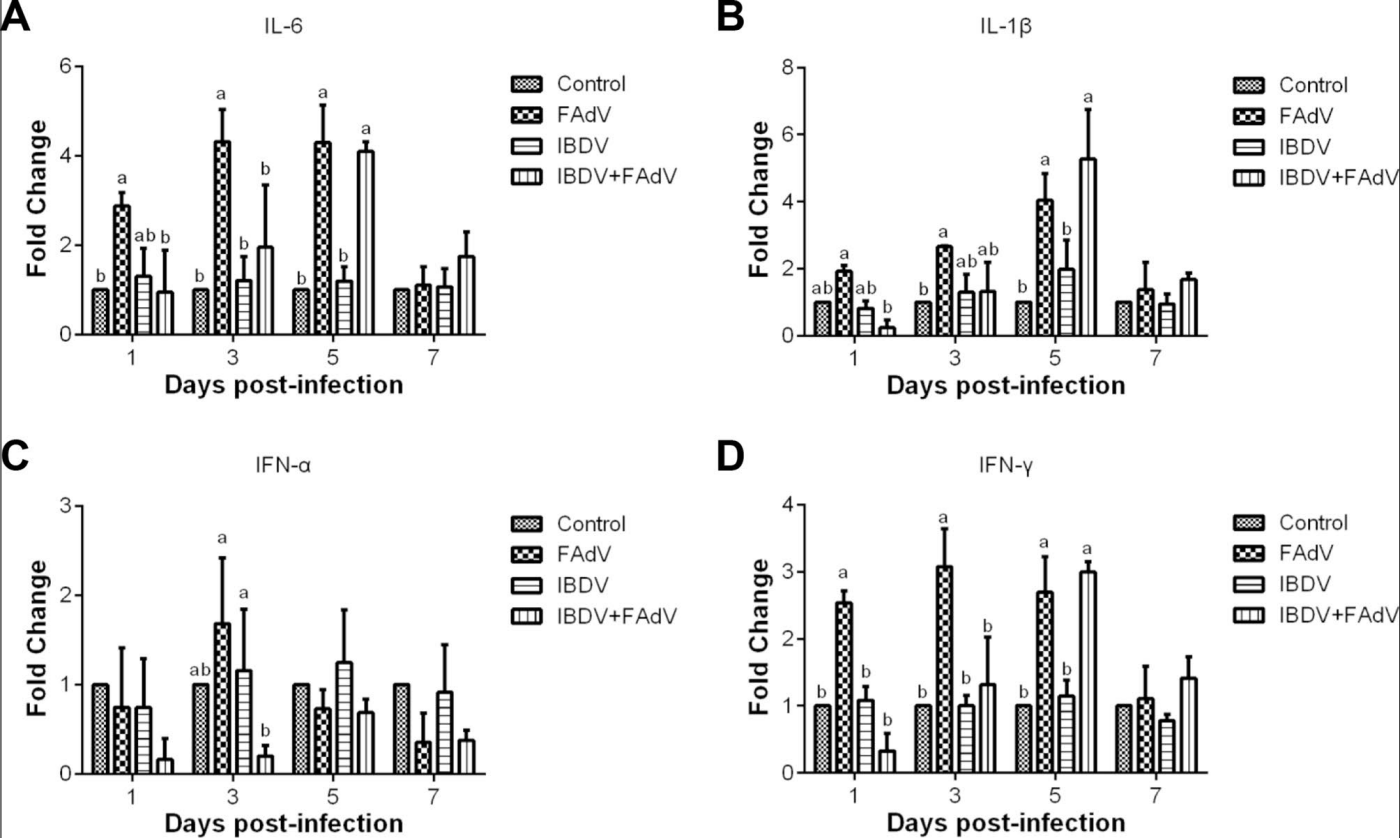
**Cytokine expression levels in the spleen of infected chickens.** Chickens were inoculated with variant IBDV-ZD-2018–1 and/or FAdV-4-HB1501, and the mRNA levels of cytokines in the spleen were measured. ^a,b^Bars with no common superscript are significantly different (*p* < 0.05).

To detect the cytokine responses induced by co-infection with IBDV and FAdV, the cytokine mRNA expression levels in the spleen were examined by using qRT-PCR. The mRNA expression levels of IL-6 in the FAdV-only group were significantly higher than those in the control group from 1 to 5 dpi with FAdV-4-HB1501 (*p* < 0.05), whereas the IL-6 mRNA expression level in the IBDV-FAdV co-infection group was significantly different from that in the control group at 5 dpi with FAdV-4-HB1501 (*p* < 0.05). Moreover, the mRNA expression levels of IL-6 in the FAdV-only group were significantly higher than those in the IBDV-FAdV co-infection group at 1 and 3 dpi with FAdV-4-HB1501 (Figure 4A; *p* < 0.05).

The IL-1β mRNA expression level increased gradually and peaked at 5 dpi with FAdV-4-HB1501 in both the FAdV-only and IBDV-FAdV co-infection groups. The IL-1β mRNA expression levels in the FAdV-only group were significantly higher than those in the control group at 3 and 5 dpi with FAdV-4-HB1501 (*p* < 0.05), while this level in the IBDV-FAdV co-infection group was considerably higher than that in the control group at 5 dpi with FAdV-4-HB1501 (*p* < 0.05). The IL-1β mRNA expression in the IBDV-FAdV co-infection group was significantly lower than those in the FAdV at 1 dpi with FAdV-4-HB1501 (Figure 4B; *p* < 0.05).

The IFN-α mRNA expression level in the FAdV-only group trended higher than that in the control group at 3 dpi, but this difference failed to reach statistical significance. These levels in the IBDV-FAdV co-infection group were lower than those in the control group at all sampling timepoints. Notably, the IFN-α mRNA expression level in the FAdV-only group was significantly higher than that in the IBDV-FAdV co-infection group at 3 dpi with FAdV-4-HB1501 (Figure 4C; *p* < 0.05). The trend of IFN-γ mRNA expression levels in the infected groups was consistent with that of the IL-6 mRNA expression levels (Figure 4D). These results demonstrate that the cytokine response in the IBDV-FAdV co-infection group was delayed in comparison with the response in the FAdV-only group.

### Biochemical index levels

**Figure 5:**
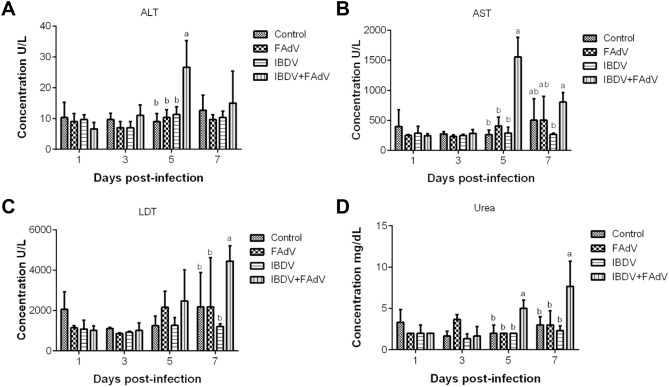
**Biochemical index levels in the serum of infected chickens.** Chickens were inoculated with variant IBDV-ZD-2018–1 and/or FAdV-4-HB1501, and the serum levels of various biochemical indices were measured. ^a,b^Bars with no common superscript are significantly different (*p* < 0.05).

The biochemical index results are presented in Figure 5. The ALT level in the serum from the IBDV-FAdV co-infection group peaked at 5 dpi with FAdV-4-HB1501 and was significantly higher than those in the other three groups at that timepoint (Figure 5A; *p* < 0.05). The serum AST level in the IBDV-FAdV co-infection group was significantly higher than those in the other three groups at 5 dpi with FAdV-4-HB1501 (Figure 5B; *p* < 0.05). The serum LDH level in the IBDV-FAdV co-infection group was significantly higher than those in the other three groups at 7 dpi with FAdV-4-HB1501 (Figure 5C; *p* < 0.05). Consistently, the serum urea levels in the IBDV-FAdV co-infection group were significantly higher than those in the other three groups from 5 to 7 dpi with FAdV-4-HB1501 (Figure 5D; *p* < 0.05).

### Antibody response

**Figure 6:**
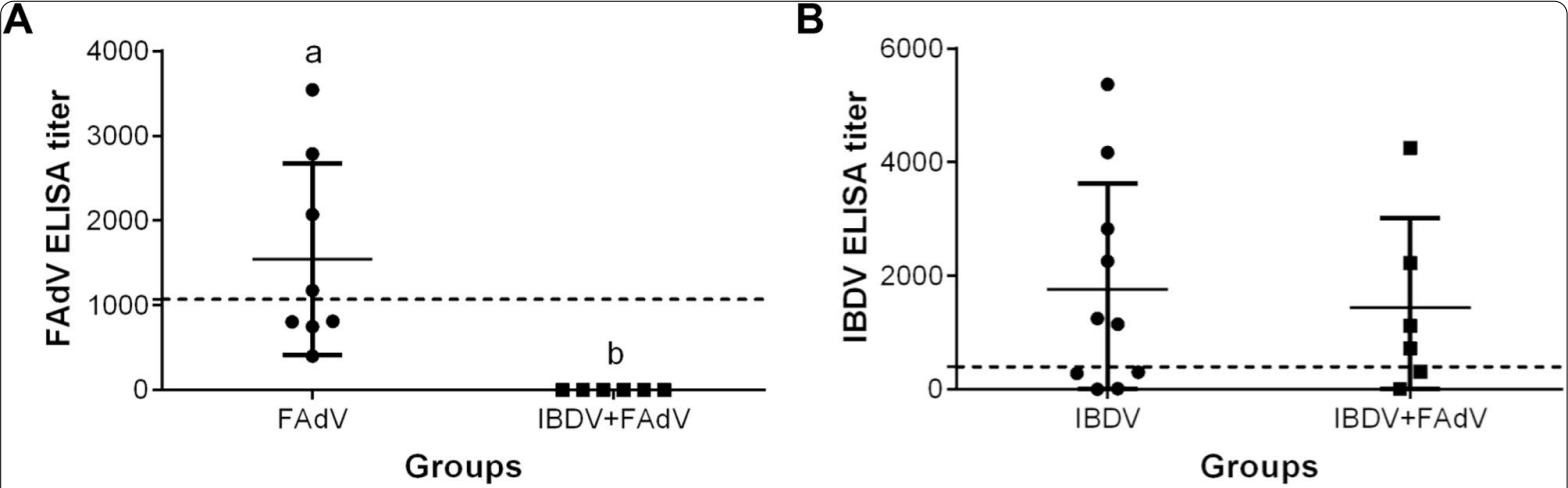
**Serum antibody responses in infected chickens.** Antibody responses in the chickens infected with variant IBDV-ZD-2018–1 and/or FAdV-4-HB1501 were determined by ELISA. **A**, **B** Cut-off titer = 1070 (**A**) or 396 (**B**). ^a,b^Bars with different superscripts are significantly different (*p* < 0.05).

The FAdV- and IBDV-specific antibody responses in chickens at 7 days post-co-infection from the infected groups are shown in Figure 6. Four out of eight chickens in the FAdV-only group had a positive anti-FAdV antibody response, whereas all chickens in the IBDV-FAdV co-infection group lacked a detectable anti-FAdV antibody response; this difference from the FAdV-only group is statistically significant (Figure 6A). The anti-IBDV antibody titers were positive in 6/10 chickens from the IBDV-only group and in 4/6 chickens from the IBDV-FAdV co-infection group. This antibody response in the IBDV-FAdV co-infection group trended lower than that in the IBDV-only group, but this difference failed to reach statistical significance (Figure 6B). As expected, chickens from the control group lacked detectable antibody responses to either FAdV or IBDV.

## Discussion

Novel antigenic variant IBDV and FAdV-4 have both occurred frequently in China, recently causing huge economic losses [6, 11], and FAdV co-infection with IBDV in chickens has been reported [16, 18]. In this study, we developed a co-infection model in SPF chickens using the novel variant IBDV strain ZD-2018-1 and the FAdV-4 isolate HB1501 to explore the interaction between these infections. Clinical symptoms, mortality, gross lesions, histopathology, tissue viral loads, cloacal viral shedding, immune-related gene mRNA expression levels, biochemical index levels, and antibody responses were evaluated.

In the IBDV-only group, the chickens showed no obvious clinical symptoms or death, but severe lesions in the BF were observed, which is consistent with previous research [7]. In the FAdV-only group, the chickens experienced a transient period of disease, and slight lesions in the liver, kidney, and thymus were found. Compared with the chickens in the FAdV-only group, those in the IBDV-FAdV co-infection group showed clinical signs earlier, their symptoms were more severe, and all of the birds in this group died. Additionally, obvious lesions in the liver, spleen, kidney, thymus, BF, and duodenum were observed in the IBDV-FAdV co-infection group chickens. The pathogenicity differences between the IBDV-FAdV co-infection group and the FAdV-only group observed here were more striking than those described by a previous report in which three or fewer chickens died in the highly virulent IBDV and IBHV co-infection group whereas no chickens died in the IBHV-only infection group [20]. These results indicate that co-infection with the novel variant IBDV and FAdV-4 could greatly enhance the clinical symptoms and tissue lesions, as well as significantly increase the mortality rate, of FAdV-4 infection in chickens.

Chickens in the IBDV-FAdV co-infection group had significantly higher viral loads in all examined tissues compared with those in the FAdV-only group, especially in the spleen and kidney at 5 dpi with FAdV-4-HB1501. Additionally, the FAdV viral loads remained at high levels from 3 to 7 dpi with FAdV-4-HB1501 in the IBDV-FAdV co-infection group. Consistent with the tissue viral loads, the cloacal viral shedding in the IBDV-FAdV co-infection group were higher than those in the FAdV-only group, and these levels remained high from 3 to 7 dpi with FAdV-4-HB1501. These findings suggest that co-infection with variant IBDV and FAdV-4 could contribute to FAdV replication in various tissues, promote viral release, and delay viral clearance [25].

To investigate the innate immune response caused by IBDV and FAdV co-infection, we examined the mRNA expression levels of IL-6, IL-1β, IFN-α, and IFN-γ in the spleen because it is the principal organ of systemic immunity [26]. IL-6 is involved in monocyte proliferation, neutrophil recruitment, and acute-phase responses [27]. IL-1β plays an important role in the acute phase of inflammation [28]. IFN-α is produced by infected cells and has immunomodulatory and antiviral ability [29]. IFN-γ is an activator of macrophages, which can inhibit viral replication through various mechanisms, such as through the induction of indoleamine 2, 3-dioxygenase, and nitric oxide synthase [30–32]. The cytokine mRNA expression levels in the FAdV-only group were significantly higher than those in the control group; this finding is consistent with previous reports [23, 33]. The cytokine mRNA expression levels in the IBDV-only group were similar to those in the control group, likely because the sampling time was over 14 days after the chickens had been inoculated with IBDV, and these infected chickens were in the recovery phase [2]. The cytokine mRNA expression levels in the IBDV-FAdV co-infection group were significantly lower than those in the FAdV-only group within 3 dpi with FAdV-4-HB1501; these levels subsequently increased greatly, and a statistically significant difference between the IBDV-FAdV co-infection and control groups could be seen at 5 dpi with FAdV-4-HB1501. These results indicate that co-infection could postpone the innate immune response in chickens, which resulted in virus replication without restraint in the early stage, subsequent induction of a cytokine storm, and eventual death. We speculate that the delayed innate immune response in IBDV-FAdV coinfected chickens may be attributed to the immunosuppression induced by lymphocyte reduction in various immune organs [34–36]. The host immune response caused by IBDV-FAdV co-infection was complex and unpredictable [37]; thus, the exact mechanism of the observations in the IBDV-FAdV co-infection group needs to be further investigated.

ALT and AST exist in hepatocytes and are released into the blood during early cell degeneration [38, 39]. LDH exists in the cytoplasm of all histiocytes in body, and the LDH content increases when tissues are injured, particularly following kidney damage [40]. Urea is an indicator of renal function, and an increase in the urea level reflects renal dysfunction [41]. The levels of ALT, AST, LDH, and urea in the serum of the IBDV-FAdV co-infection group were all higher than those in the other three groups, indicating that IBDV and FAdV co-infection can damage the functions of the liver and kidney whereas infection with FAdV alone cannot. Given that liver and renal dysfunction are typical clinical manifestations of FAdV infection [12, 42], these results reconfirm that IBDV and FAdV co-infection can enhance the pathogenicity of FAdV infection.

Serum antibodies have the greatest impact on systemic virus replication and viremia [43]. In this study, only 60% of the investigated birds from IBDV-only and IBDV-FAdV groups have developed specific antibodies against IBDV at 21 dpi. This result may relate to the severe immunosuppression in birds and the mismatched IBD ELISA kit (IDEXX). But the bursa of Fabricius in each IBDV infected chicken was atrophy at 28 dpi, which confirmed that the IBDV successfully infected the birds. The anti-FAdV antibody titers in chickens from the IBDV-FAdV co-infection group were significantly lower than those in the FAdV-only group. Furthermore, all the IBDV-FAdV co-infection group chickens lacked an anti-FAdV antibody response, indicating that the humoral response to FAdV was suppressed [7], which led to unrestrained FAdV replication and spread. The impaired humoral response likely resulted from damage to the BF, which is the central immune organ for the generation of diverse antibody in young chickens [34]. These findings highlight that the humoral response immunosuppression caused by the variant IBDV can have important consequences.

Notably, the pathogenicity of FAdV-4-HB1501 depends on the age of infected chickens, route of inoculation, and timing of infection. In our previous research [21], 3-week-old SPF chickens infected with 10^6^ TCID_50_ FAdV-4-HB1501 via the oral route did not show any clinical symptoms, whereas those inoculated intramuscularly or intravenously with the same dose had mortality rates of > 70%. Additionally, no clinical signs were observed when 3-week-old SPF chickens were simultaneously coinfected with IBDV-ZD-2018-1 and FAdV-4-HB1501 by the oral route. These findings indicate that the pathogenicity of FAdV is probably related to mucosal immunity. Thus, the enhancement of FAdV pathogenicity by IBDV co-infection may be a consequence not only of innate and adaptive immune response inhibition but also of the mucosal immunity damage in IBDV-infected chickens [44, 45], although this possibility requires further investigation. co-infection involves complex interactions between infectious agents, host, and environments [46]; despite the associated challenges, future studies need to be performed to fully understood the role of co-infections in poultry.

In conclusion, co-infection with variant IBDV-ZD-2018-1 and FAdV-4-HB1501 could induce severe immunosuppression and enhance the pathogenicity of FAdV-4 in chickens. Therefore, more attention should be paid to co-infections in poultry.

## Abbreviations

AIV: Avian influenza virus
ALT: Alanine aminotransferase
AST: Aspartate aminotransferase
BF: Bursa of Fabricius
CIAV: Chicken infectious anemia virus
CID_50_: The median chicken infectious dose
dpi: Days post-infection
FAdV: Fowl adenovirus
FAdV-4: Fowl adenovirus serotype 4
IBD: Infectious bursal disease
IBDV: Infectious bursal disease virus
IBHV: Inclusion body hepatitis virus
LDH: Lactate dehydrogenase
LMH: Leghorn hepatoma cell line
SPF: Specific pathogen-free
TCID_50_: 50% Tissue culture infection dose
